# The In Vitro, Ex Vivo, and In Vivo Effect of Edible Oils: A Review on Cell Interactions

**DOI:** 10.3390/pharmaceutics15030869

**Published:** 2023-03-08

**Authors:** Ioannis Tsamesidis, Eleni P. Kalogianni

**Affiliations:** Department of Food Science and Technology, Sindos Campus, International Hellenic University, 57400 Thessaloniki, Greece

**Keywords:** cytotoxicity, fish oils, vegetable oils, seed oils, edible oils, oxidative stress biomarkers, antioxidant properties, in vitro models, ex vivo, in vivo studies

## Abstract

Consumption of edible oils is a significant part of the dietary pattern in the developed and developing world. Marine and vegetable oils are assumed to be part of a healthy food pattern, especially if one takes into account their potential role in protecting against inflammation, cardiovascular disease, and metabolic syndrome due to the presence of polyunsaturated fatty acids and minor bioactive compounds. Exploring the potential effect of edible fats and oils on health and chronic diseases is an emerging field worldwide. This study reviews the current knowledge of the in vitro, ex vivo, and in vivo effect of edible oils in contact with various cell types and aims to demonstrate which nutritional and bioactive components of a variety of edible oils present biocompatibility, antimicrobial properties, antitumor activity, anti-angiogenic activity, and antioxidant activity. Through this review, a wide variety of cell interactions with edible oils and their potential to counteract oxidative stress in pathological conditions are presented as well. Moreover, the gaps in current knowledge are also highlighted, and future perspectives on edible oils and their health benefits and potential to counteract a wide variety of diseases through possible molecular mechanisms are also discussed.

## 1. Introduction

Edible fats and oils are an indispensable nutritional resource for human health as they possess complex properties; thus, it is recommended that oils and fats be consumed as part of a healthy diet [1]. According to the Food and Drug Administrationin the US (FDA), dietary supplements (fish oil capsules, etc.) can help people improve or maintain their overall health. However, they may also come with health risks, and for this reason it is important to know the facts before deciding to take any dietary supplement. Furthermore, regarding food, nutraceuticals, and drugs, the FDA is responsible for protecting public health by regulating human drugs and food in the US, whereas the European Food Safety Authority (EFSA) is responsible for animal and human health and welfare in Europe. There is a class of food products that scientific experts deem to mention as “generally recognized as safe” (GRAS) for their intended circumstances of use, but which are not subject to FDA premarket approval. The FDA has a voluntary notification process under which a manufacturer may submit a conclusion that the use of an ingredient is GRAS [2]. On the other hand, the EFSA is more demanding regarding novel foods and novel ingredients, and this applies also to lipids from uncommon ones to human consumption sources. 

Another very important aspect related to food quality is food production. Production of safe and high quality food is the aim of the food industry [3]. Food processing, a crucial link between production and consumption within the food value chain, entails the conversion of raw materials into edible, safe, and organoleptically and culturally acceptable food products [3]. Ex vivo and in vitro biological studies highlight the significance of key sustainability indicators and impact evaluation when creating instruments to study how human cells interact with the constituents of edible oils [1,4,5,6,7]. These methods offer less expensive means to survey food components and reduce the probability of surprises when clinical trials are performed to elucidate their health benefits. For the aforementioned reasons, in vitro assays for food applications are performed in human cells and mice models, such as hepatocytes [4,8], whole blood cells [5,9], neural cells [10,11], and various other cell types (keratinocytes and smooth muscle cells) [6,7] and mice/rat models, such as C57BL/6, Swiss strain mice, and Sprague Dawley rats [12,13,14], etc., to investigate edible oils’ “natural” health effects.

The biological assays are considered to be the analysis of the antibacterial [15,16], anti-inflammatory [17,18,19,20], and antiviral properties [14], as well as the antitumor activity [21], as these safe alternative assays mimic cell interaction in vitro when investigating the biological properties of treatment due to their “natural” status. We will focus on the cell activity of three well-known oil categories, namely (i) vegetable oils, (ii) essential seed oils, and (iii) fish oils. Many plants contain extractable oils that have, for centuries, been used either as food, in cosmetic formulations, or for their health benefits [22,23]. Oleaginous seeds, nuts or kernels, or oleaginous fruit pulps have an oil content reaching or exceeding in some cases 40% of their total weight [24]. Medicinal plants and their oil extracts are widely examined in vitro for their biological properties [25]. There are a variety of methods used for the extraction of essential oils, with each method exhibiting certain advantages and determining the biological and physicochemical properties of the extracted oils [26]. On the other hand, fish oils (FO) are considered to be effective for the prevention and the management of pathological diseases, such as dyslipidemias [27], cardiovascular disease (atrial fibrillation, atherosclerosis, thrombosis, inflammation, and sudden cardiac death, among others), diabetes, cancer, depression, and various mental illnesses, age-related cognitive decline, periodontal disease, and rheumatoid arthritis [28,29,30,31,32]. The great potential of fish oils to counteract and manage such disorders is due to their rich content in terms of fatty acids, including polyunsaturated fatty acid (PUFAs), docosahexaenoic acid (DHA) and eicosapentaenoic acid (EPA), which are long-chain omega-3 fatty acids [33]. The observations linking the Eskimos’ high dietary consumption of fish oils to a very low incidence of inflammatory illnesses and ischemic heart conditions sparked interest in fish oils [19]. The ability of fish oils to reduce the metabolic activity of cells has been well documented in the literature. These abilities include inhibiting quorum sensing, destroying or inactivating genetic material, changing the pH gradient across the cytoplasmic membrane, and influencing the phospholipid bilayer of the cell membrane [34]. However, they do not cause cell death, and different cell models were studied during the last few years to confirm their biocompatibility and evaluate their antioxidant activity [14,27]. 

The available literature provides only limited data about the complete mode of action of vegetable oil, essential oil, and fish oil biocompatibility and on the oxidative stress conditions with a wide variety of cell types. As such, in order to analyze the oxidative stress conditions in cells, reactive oxygen species (ROS) should be determined. In parallel with cellular aerobic respiration, ROS, which are both radical and non-radical chemical species generated by the partial reduction of oxygen, accumulate physiologically. ROS can cause cellular death and DNA damage. Superoxide dismutase, catalase, glutathione peroxidase, and albumin are just a few of the specific enzymes and thiolic antioxidants that cells have available to them as ROS defense mechanisms. Cells also produce biologically active metabolites which are crucial to the physiological resolution of the inflammatory process. The food may support these processes by supplying macronutrients, such as omega-3 fatty acids, which are substrates for the manufacture of resolution mediators, as well as micronutrients, such as vitamin C and vitamins A and E, which can neutralize ROS [20,35]. 

The present article provides an overview of the state of knowledge regarding the effects of edible oils on various cell types in in vitro, ex vivo, and in vivo conditions. It presents which nutritive and bioactive components of various edible oils show biocompatibility, as well as any oxidative stress conditions and/or antimicrobial/antitumor properties which may reveal their potential molecular mechanisms. The in-depth literature analysis will present how fatty acids affect biochemical pathways following cell contacts, as well as how they counteract oxidative stress.

## 2. Materials and Methods

**Figure 1:**
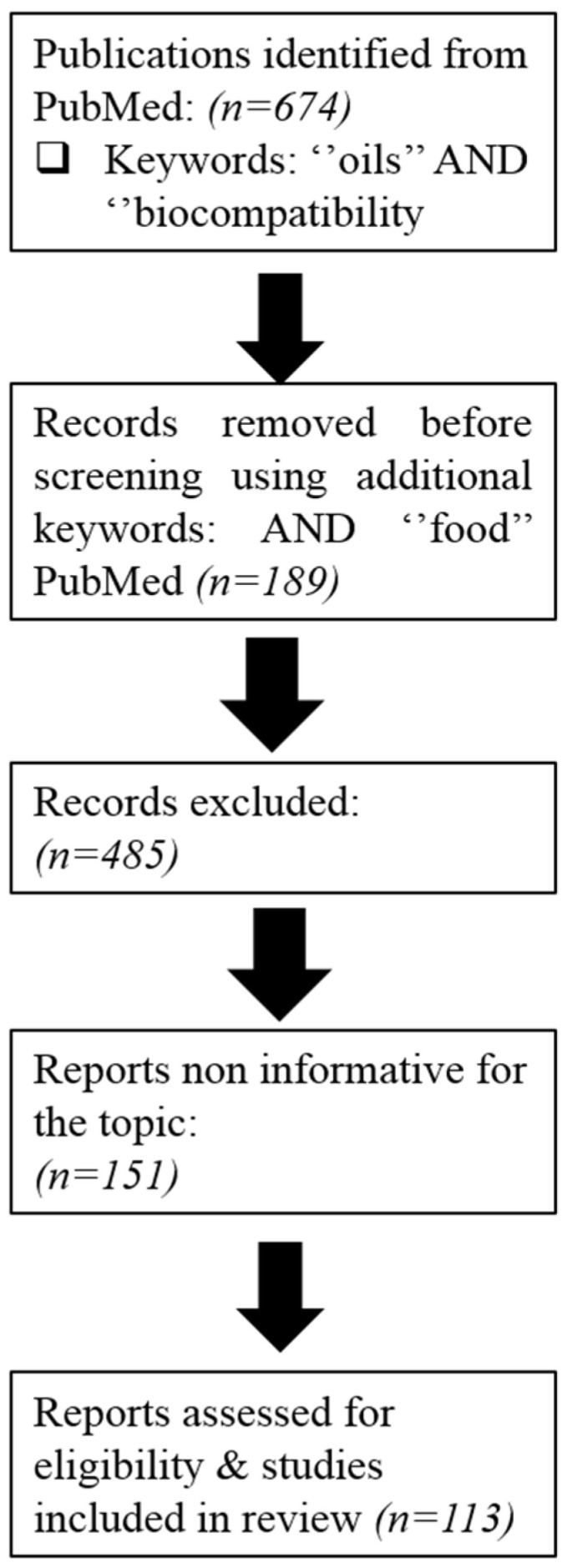
Flow diagram of the screen methodology for Table 1.

The literature review was conducted using the following pre-specified search terms: edible oils and biocompatibility. Figure 1 presents the screening methodology used to identify and select the relevant research articles for this review.

### 2.1. Search Strategy

An electronic literature search without language restrictions was conducted until 11 February 2023. For the search strategy, the following databases were used: (i) Scopus^®^ (Elsevier), (ii) PubMed^®^ (NLM: United States National Library of Medicine), and (iii) Web of Science. The search criteria included the following two main keywords: “edible oil”, and “biocompatibility”. In a second step, the results from the aforementioned searching criteria were limited to those including the following keyword: “human consumption”. Regarding Table 2 and the role of fatty acids in cell interactions, the keywords used were “fatty acids”, and “cell functions”. For Table 3, the keywords used were “fish oil supplements” “vegetable oils” “οxidative stress” and “cell interactions”. Moreover, to enrich our selection of the literature with more studies, the references of individual papers were also examined. The aim of this review was to discuss applications of edible oils in cell interactions in vitro, ex vivo, and in terms of clinical outcomes derived from in vivo models and clinical studies. Hand-searching from selected review articles and other included articles was also performed.

### 2.2. Study Selection/Inclusion Criteria

All the studies included in this review fulfilled the following criteria: (1) biocompatibility assays related to the toxic behavior of foods; (2) dietary oils including vegetable oils, fish oils animal fats and essential oils were primarily investigated; (3) cell types without restrictions; (4) the biochemical mechanism of cells after interactions with oils.

### 2.3. Data Extraction and Outcomes

Data were extracted and entered by two authors (I.T and E.P.K), while the second author (E.P.K) was responsible for verifying the information. All possible discrepancies were resolved by agreement.

## 3. Results and Discussion

### 3.1. Effect of Edible Oils: In Vitro, Ex Vivo and In Vivo Studies 

A biocompatibility assay is the most used method to describe the appropriate biological requirements of newly introduced compounds, foods, and materials for food technology and biomedical applications by conducting in vitro experiments. Table 1 summarizes the in vitro, ex vivo, and in vivo effect of edible oils. The cell lines used by researchers for the in vitro analysis are Vero cells [36], hepatocytes [4,8], whole blood cells [4,5,9], and cancer cell lines, such as Jurkat and HeLa cells [37,38,39]. Few studies were performed in ex vivo conditions [40,41], referring to experimentation or measurements performed in or on cells/tissues in an artificial environment outside the organism with minimal alteration of natural conditions. In the in vivo studies, the effects of various biological entities are tested on whole living organisms (animals, including humans, and plants). In this contribution, we review the different preclinical models (in vitro, ex vivo, and in vivo) from their development to application for studying the effect of edible oils in contact with various cell types [5,37,42]. In this section, in vitro, ex vivo, and in vivo studies referring to the oil-effect in cells will be discussed. All edible oils included in this review are conventional edible oils produced for human consumption. 

**Table 1:** In vitro, ex vivo and in vivo models used for the examination of the effects of different oils on cells.

	Cell Category	Oil Types	Model	Study	Application and Key Findings	References
Vegetable/Seed oils	Chicken embryo fibroblasts	Lavender oil	Chicken	In vitro	Effect on wound-healing: enhances the regeneration of new tissue and defense against bacteria	[43]
	*Candida albicans*	Melaleuca oil	Fungi	In vitro	Superior antifungal activity of melaleuca oil in comparison to fluconazole	[44]
	Pigment epithelium cells and mice	Olive oil, corn oil, argan oil, and camelina oil	Human and mice	In vitro and in vivo	In vitro cytotoxicity: cytocompatibility of vegetable oils with epithelium cells	[45]
	Conjunctival cells	Olive, camelina, *Aleurites moluccana*, maize oils, castor oil	Human	In vitro	In vitro cytotoxicity: lack of cytotoxicity for all the tested oils	[46]
	Keratinocyte cells	Red raspberry seed oil	Human	In vitro cytotoxicity	Safety and efficacy assessment: biocompatible with antioxidant activity	[6]
	Mesenchymal stem cells (MSCs)	Olive oil	Human	Proliferation, regeneration capacity	Olive oils affect MSC maintenance and differentiation	[47]
	Immortalized human gingival fibroblasts (HGF)	Ozonized olive oil	Human	Cytotoxicity evaluation	Cytocompatibility of the ozonized olive oil as an alternative antibacterial agent	[48]
	Human melanoma cells	Extra virgin olive oil	Human	Cell viability assay	The ability of extra virgin oil to counteract the proliferation of cutaneous melanoma cells	[49]
	CCD-1064Sk fibroblast line	Olive oil	Human	Proliferation and antimicrobial properties	Inhibiting the growth of bacteria strains	[50]
	Cultured malignant cells	Flaxseed oil	Human	Cell viability	Induction of apoptosis in malignant cancer cells	[51]
	Caco-2 cells	Sunflower seed oil	Human	In vitro cytotoxicity	Cell damage of Caco-2 colon cancer cells	[52]
	-	Coconut oil and sunflower oil	Human	Clinical trial	Triacylglycerol, LDL, and VLDL cholesterol levels were higher in the diabetic subjects compared to the controls.	[53]
	-	Virgin coconut oil (VCO)	Human	Open label, randomized, controlled, crossover study	Daily VCO intake significantly increased high-density lipoprotein cholesterol	[54]
	-	Coconut oil, olive oil	Human	Clinical trial	Coconut oil did not significantly differ from olive oil for TC/HDL-C and non-HDL-C	[55]
Essential oils	Vero cells	Myrtaceae essential oils (cajuput oil, clove oil, kanuka oil, and manuka oil)	Human	In vitro cytotoxicity	Anticancer properties	[36]
	Hepatocytes and erythrocytes	Eygenol, thymol, menthol	Rat	In vitro cytotoxicity	Essential oils may cause periapical tissue injury by causing membrane lysis and surface activity.	[4]
	Whole blood cells	Essential oils of *Thymus* and *Origanum* plants	Human	In vitro cytotoxicity to investigate the genetic, oxidative, and cytotoxic effects of thymol in cultured human blood cells	Enhanced biocompatibility with human cell lines and an inhibitory effect on the production of biofilms.	[5,16]
	Jurkat, J774A.1, and HeLa cells lines	Essential oils of *Eucalyptus benthamii*	Human	Inhibitory effect	Anticancer activity, decrease in cell DNA	[37]
	Ovarian cancer cell and foreskin fibroblasts	*Linum usitatissimum* seed essential oil	Human	Cell viability	Apoptotic activity anti-angiogenic activity	[56]
	*Candida Albicans*	Cumin seeds essential oil	Bacteria	Antibacterial activity	Effective against candidiasis	[57]
	A. Salina	Eugenol and garlic essential oils	Aquatic crustaceans	Acute toxicity test with A. salina	Bactericidal activity against fish pathogenic bacteria	[58]
	Pig tracheal epithelial cell line NPTr and porcine respiratory bacterial pathogens	Essential oils from *Abies balsamea*, *Cinnamomum verum; Coriandrum sativum, Ledum groenlandicum, Mentha piperita, Salvia officinalis, Origanum majorana, Thymus vulgaris*, and *Satureja montana*	Pig and bacteria	In vitro	In vitro cytotoxicity and antibacterial activity, synergistic growth inhibition of *S.Suis* from the tested oils	[15]
	Skin and lung cells	Essential oils from *Abies koreana, Platycladus orientalis*	Human	In vitro	Cell viability assay, plant essential oils can be used safely	[59]
	Oxyntopeptic cells and somatostatin and ghrelin immunoreactive cells	Essential oils from thyme, cinnamon, and rosemary	Fish	In vitro	Na^+^K^+^-ATPase expression was modified in gastric mucosa	[60]
	K562 cells	Essential oils	Human	In vitro	Telomerase (hTERT) gene transcription was not increased by telomere-protective oils.	[61]
	*E. coli, K. pneumonia, St. aureus*	Flower oil, bud oil	Bacteria	In vitro	Antibacterial properties: flower oils presented higher antimicrobial effects against *K. pneumoniae*	[62]
	Bacteria in humans	Organic olive oil-based denture adhesive	Human	Clinical trial	Inhibition capacity for the growth of *Candida albicans*	[63]
Marine oil	Smooth muscle cells (SMC)	Fish oil	Human	In vitro cytotoxicity assay	*SMC cells resist apoptosis with fish oil*	[7]
	*Bacillus cereus*	Marine oil spills	Bacteria	In vitro cytotoxicity assay	Inhibition of bacterial growth	[64]
	Epithelial cells Cancer cells	Bottarga extracts	Human	In vitro cytotoxicity activity	Inhibition of cancer cell growth	[65]

#### 3.1.1. Vegetable and Essential Oils

##### Human Consumption

Vegetable oils, a major source of edible lipids, are an indispensable component of human diet, and appear to protect body tissues carrying liposoluble vitamins and containing a significant source of fatty acids, especially those essential fatty acids. Essential fatty acids (the omega-3 family α-linolenic acid and the omega-6 linoleic acid) constitute building blocks of longer chain fatty acids and have the role of mediants that regulate biological processes but are not synthesized endogenously and, therefore, have to be obtained in the diet [66]. Furthermore, to provide healthy food and, consequently, to create good quality food, especially including high quality vegetable oil, is one of society’s major challenges in terms of diet [67].

Within this context, individual fatty acids from vegetable oils, as well as oil-soluble bioactive compounds, may play a different role in human health, especially for the management of acute and chronic diseases, such as cardiovascular disease, breast cancer, and inflammation [68,69,70,71]. One of the most examined oils for its health properties is olive oil [18,68,69,71,72]. Olive oil is rich in monounsaturated oleic acid and phenolic compounds, as well as squalene [73,74,75]. The capacity of olive oil to stop or slow down the inflammatory processes linked to chronic degenerative disorders also supports its role as an anti-atherosclerotic, improving the lipid profile too. Human consumption of sesame oil, an oil rich in linoleic and oleic acids, as well phenolic antioxidants, and extracted from sesame seeds, was used in clinical trials for patients with high CVD risk and presented an effective natural cardiovascular therapeutic potential [76]. Another study presented the positive effect of pomegranate seed oil, a natural source of conjugated linoleic and linoleic acid, on hyperlipidemic subjects, concluding that their daily administration for a period of a month could improve the lipid profile of the subjects [77]. Lately, the effects of *Nigella sativa* L. seed oil from Nigeria on abnormal semen quality in infertile men was investigated. The results of Nigella seed oil were impressive, indicating that daily intake of sativa oil was able to improve abnormal semen quality in infertile men [78]. Furthermore, as regards coconut oil, a source of saturated medium chain fatty acids, various clinical trials showed that it increased blood lipids, high density lipoproteins (HDL), and cholesterol indicating the risks in health [54,55,79,80]. Another study in cardiovascular disease (CVD) patients indicated that cooking with coconut oil instead of sunflower oil did not affect the lipid and oxidative profile or the CVD risk factors and events related to lipids in patients receiving normal medical care [53]. In conclusion, a recent meta-analysis of the impact of coconut oil finds that, compared to other oils, consuming coconut oil considerably raises blood levels of total cholesterol and lipids [81].

##### In Vitro and Animal Studies

In vitro studies related to vegetable oils analyzed the beneficial role of olive oil to alter the physiology of mesenchymal stem cells (MSCs) by reducing apoptosis, oxidative stress, and the inflammatory status of MSCs [47]. Moreover, the ability of olive oil to inhibit melanoma cells through the transcriptional modulation of relevant genes and microRNAs was achieved, suggesting the anticancer properties of olive oil [49]. The vast majority of the studies indicate the protective role of olive oil against cancer cells, suggesting it as a promising agent for cancer risk reduction [82]. Furthermore, experimental in vivo studies have demonstrated a preventive effect of olive oil and its constituents on mammary carcinogenesis [83]. The beneficial effect of olive oil was explained by a variety of intricate and interconnected mechanisms, including modifications to the transcriptome, protein expression, and epigenetics in cells that affect a number of signaling pathways. The same anticancer properties were also observed when sunflower seed oil was used in caco-2 cancer cells, indicating its chemopreventive role [52]. Regarding allergic symptoms, only olive oil in mice models appeared to offer an improvement effect on clinical allergic symptoms of the in vivo allergy tests [84]. Other vegetable/seed oils, such as flaxseed oil, enhanced the cell death of cancer cells disrupting the mitochondrial function of the malignant cells [51]. 

P. Schnitzler and his team, who were pioneers in the field, studied the effects of myrtaceae essential oils (cajuput oil, clove oil, kanuka oil, and manuka oil) on hepatocytes and red blood cells to comprehend the mechanism of cell membranes in the late 1980s. They identified biochemical modifications, such as lactate dehydrogenase (LDH) leakage, related to the periapical tissue damage produced by essential oils and consequent membrane lysis, surface activity and membrane affinity, and lipid solubility. Biocompatibility assays were also performed to reveal the toxicity of components against cancer cell lines or pathogens. In detail, the role of the essential oil of *Eucalyptus benthamii* against Jurkat, J774A.1, and HeLa cells lines was investigated [37]. Regarding its cytotoxic activity, they used a common MTT(3-[4,5-dimethylthiazol-2-yl]-2,5 diphenyl tetrazolium bromide assay), presenting cytotoxic effects against Jurkat cells while also measuring LDH activity and DNA damage to explain the cell death by apoptosis. Furthermore, the mentioned essential oils from the leaves of E. benthamii presented cytotoxicity against the investigated tumor cell lines, which confirms their antitumor potential [37]. The same set of experiments in in vitro test systems of MTT, DNA damage, and LDH activity was performed using essential oils of *Thymus* and *Origanum* plants in whole blood cells [9]. The aim of this study was to investigate the potential of these essential oils to be used as therapeutic antioxidants against different diseases. Antioxidants were statistically increased, while ROS levels were decreased. According to the results of the LDH assay, thymus essential oil induced cytotoxicity on cultured human blood cells in a time- and dose-dependent manner [9]. 

##### Antimicrobial Properties

Regarding the antimicrobial properties of vegetable oils, the cytocompatibility of human gingival fibroblasts with ozonized olive oil was assessed in order to be considered as an alternative antibacterial agent [48,85]. Further research showed that the phenolic components of olive oil, such as luteolin, apigenin, ferulic, coumaric acid, or caffeic acid, have antibacterial effects and promote the regeneration of fibroblasts [50]. More studies on antimicrobial properties have been dedicated to the essential oils. Essential oils target the lipids of the cell membranes of bacteria, disrupting the cell wall structures and the membrane permeability, presenting both single and multiple target activities. For this reason, essential oils could be used in combination with antibiotics (oral consumption) and their antimicrobial screening and evaluation methods are under investigation. Well diffusion, disk diffusion, and broth or agar dilution are a few well-known and often used bioassays. The estimation of minimum bactericidal concentration (MBC), which is defined as the concentration killing 99.9% or more of the initial inoculums, is the method used to estimate bactericidal activity most frequently. The antibacterial activity of various essential oils was investigated, such as cumin seed essential oil [57] and melaleuca oil [44]. The aforementioned oils demonstrated antibacterial activity against cariogenic *Streptococcus mutans*, *Lactobacillus acidophilus*, and commensal *Streptococcus sanguinis*, with potent antimicrobial properties. The combination of antioxidant and antibacterial activities of all the essential oil formulations appeared useful for the antimicrobial treatment. Regarding flower oils, the essential oil extracted from magnolia flowers showed higher antimicrobial efficacy against *Klebsiella pneumoniae* and *Staphylococcus aureus* [62]. 

#### 3.1.2. Fish Oils

##### Human/Animal Consumption

The application of new formulations of fish oils in the food industry has attracted considerable interest due to their high biocompatibility and enhanced multifunctional performances compared to conventional additives. The health benefits of fish oils have been well established, and a variety of cell types were investigated. Perales et al. presented both in vitro in smooth muscle cells (SMCs) and in vivo in chicks that replacement of a cholesterol-rich diet with a fish oil-rich diet can produce some reversal of the cholesterol-induced changes, increasing the resistance of SMCs to apoptosis [7]. Furthermore, bottarga oil incubated with epithelial cells presented significant changes in fatty acid composition but not in cholesterol levels, indicating the beneficial role of bottarga oil in these cells [65]. Other animal models used in in vivo studies were rats, i.e., Wistar, omega 3 (n-3)-depleted rats, following or not following a hypercholesterolemic diet with or without asthma [27,86,87,88].

##### Other Properties

In vitro studies investigating the effect of fish oils used the following cell lines: neural cells [11,89], and white blood cells [90,91,92]. Platelets were also investigated after interacting with fish oil supplementation, as well as their potentially increased risk of bleeding, presenting no effect to platelet aggregation [93]. Another set of immune cell experiments involved nine healthy volunteers who consumed 18 g/day of fish-oil concentrate rich in n-3 polyunsaturated fatty acids, which resulted in the in vitro production of interleukin-2, suggesting that the effect of dietary n-3 fatty acids in some diseases may be partially mediated by decreased production of interleukin-2 and decreased mononuclear cell proliferation.

### 3.2. Effect of Fatty Acids in Biochemical Pathways after Cells Interactions

A balanced diet is essential for the correct function of our organism and the cell responses to counteract pathogens. Our immune system is the cell line that defends our body, and many dietary components, including macronutrients, such as fatty acids and micronutrients, such as vitamin D, have been proven to have immuno-regulatory qualities. Metabolites produced from omega-3 and omega-6 have a significant role in immunological regulation [54]. These metabolites can be classified into many groups and are commonly referred to as pro-resolving mediators (SPMs). Since fatty acids (FAs) can be stored as triglycerides, destroyed by oxidation, or utilized in the formation of phospholipids, the primary constituent of cellular membranes, they are frequently linked to structural and metabolic roles [66]. It has been demonstrated that these lipids act as regulators in several cell types. FAs can act as secondary messengers, enzymatic activity regulators, and substrates to produce cytokines. Table 2 summarizes the effect of the most potent fatty acids included in edible oils, especially in fish oils, to beneficially interact with our cells.

**Table 2:** Biochemical role of oils rich in specific fatty acids.

	Oil	Characteristic Fatty Acids	Cell Category	Effect	Study	References
Marine oils	Fish oil	Omega-3 Fatty Acids	Macrophages	Reduce inflammation, regulate production of cytokines	In vitro	[90]
	Fish oil	Omega-3 Fatty Acids	Neutrophil	Omega-3-derived metabolites inhibited migration of neutrophils	In vitro	[91]
	Fish oil	EPA (eicosapentaenoic fatty acid)	T-Cells	Suppressive effect of dietary omega-3 on T cell function	In vitro	[92,94]
	Fish oil	Omega-6 fatty acids	*E. Coli*	No effect on phagocytic capacity	In vitro and in vivo	[95]
	Carp oil	Oleic acid	Female 5-wk-old C57BL/6 strain mice	Tumor growth inhibition	In vitro	[12]
	Fish oil and corn oil	Omega-3 and omega-6 fatty acids	Sprague Dawley rats	Reduction in PM_2.5_-induction in the lung and systemic inflammatory responses	In vivo	[96]
	Fish oil	N-3 long chain polyunsaturated fatty acids (LCPUFAs) (EPA, DHA)	Ex vivo in man	Increased oxidation of LDL	Ex vivo pilot study	[40]
	Fish oil	Omega-3 and omega-6 fatty acids	Adult male Sprague-Dawley rats	On testicular steroidogenesis, adipokine network, cytokines, and oxidative stress in adult male rats	In vivo	[97]
	Fish oil	ω-3 PUFAs, EPA, and DHA	Wistar rats	Reduction in oxidative stress and inflammation markers	In vivo	[14]
Essential oil	Essential oils	Capric acid, lauric acid	Oxyntopeptic cells and somatostatin and ghrelin immunoreactive cells	The effect of feed supplemented with essential oils (EOs) on the histological features in sea bass’ gastric mucosa and the increase in the number of cells in the essential oil diet	In vitro	[60]
Vegetable/seed oil	Olive oil	Swiss mice	N-9 MUFA, oleic acid, and phenolic compounds	Wound healing	In vivo	[13]
	Palm oil	C57BL/6J mice	Palmitic acid	Metastasis	In vivo	[98]
	Sunflower oil or soybean oil	Rabbits	Omega-6 fatty acids	Folliculogenesis	In vivo	[99]
	Virgin olive oil	Pancreatic cells of rats	Monounsaturated fatty acids	Anti-inflammatory properties	In vivo	[72]

#### 3.2.1. Fish Oil

##### Human/Animal Consumption 

From the early 1990s, well-structured clinical trials started to take place in hospitals to analyze the potential of omega-3 fatty acids against different pathologies. The effect of omega-3 fatty acids in patients undergoing coronary artery bypass graft Surgery [67,68], HIV-seropositive patients [69,70,71], patients with multiple sclerosis [72,73], children with attention deficit hyperactivity disorder [18], patients with Alzheimer’s disease [27,74], patients with prostate cancer [75], and patients on hemodialysis [76,77], was investigated. The administration of vitamins or n-3 (PUFAs) in patients undergoing coronary artery bypass graft (CABG) surgery attenuated post-operative oxidative stress during CABG surgery [100]. Beneficial effects were observed in the above studies for most of the patients but without underlying any specific biochemical pathway, except the oxidative stress alteration that will be discussed in the last section.

On the other hand, in animal models, Enders and his team investigated the potential of dietary supplementation with omega-3 fatty acids to suppress interleukin-2 production and mononuclear cell proliferation. The experimentation procedure comprised of different time points of blood collection. The results suggested that the effect of dietary omega-3 fatty acids in macrophages was notable, especially to their proliferation rate [52]. In another study, neutrophils’ proliferation rate was investigated after a rich fish oil diet in human subjects. There was no effect on neutrophil aggregation, and the fish oil supplementation boosted neutrophil EPA concentration from undetectable levels. In fact, it was demonstrated that supplementation with omega-3 fatty acids has positive effects on several T-mediated illnesses, including autoimmune hepatitis and asthma [78]. However, the modulatory effect of omega-3 fatty acids differed for each phenotypic subgroup of T cells. In conclusion, the interpretation of the results appeared most fruitful in animal models in comparison to the human clinical trials. Human clinical trials demonstrated the beneficial effect without providing in depth details for the affected molecular pathway.

#### 3.2.2. Vegetable, Essential Oils Supplements

##### Human/Animal Consumption

In vivo studies related to wound healing properties revealed the potential effect of olive oil. In details, Swiss mice under stress-induced conditions presented an increase in the expression of vascular endothelial growth factor and in the numbers of macrophages and neutrophils indicating increased inflammation levels. Consumption of olive oil containing n-9 monounsaturated fatty acids (MUFA), oleic acid, and phenolic compounds was able to reverse stress-induced increase in catecholamine levels and oxidative damage. The epinephrine-induced decrease in fibroblast migration and collagen deposition and the rise in lipid peroxidation were all reversed by olive oil therapy. In conclusion, supplementing mice with olive oil but not with fish oil speeds up the healing of cutaneous wounds [13]. Moreover, the effect of dietary fatty acids from extra virgin olive oil (EVOO) were tested in rats with induced pancreatitis to discriminate the anti-inflammatory effect of the EVOO. Interestingly, the inflammatory parameters were modulated, and the disease progression was interrupted. Furthermore, the reproductive performance of rabbits was improved due to the (PUFA)-enriched diet [99]. On the other hand, palmitic acid derived from palm lung metastasis of melanomas, indicating the increased proliferation rate of the cells [98]. Furthermore, carcinogenesis and the role of fatty acids was investigated in female rats after consumption of sunflower, rapeseed, olive, or coconut oil. Interestingly, a statistically significant higher adduct levels in various organs in the sunflower diet group was observed, which was associated with a higher risk of genotoxic cancer. Evidently, a reduction in adduct levels was not significantly influenced by the vegetable oils’ vitamin E concentration. Rapeseed-oil feeding had no discernible influence on the levels of pro-carcinogenic DNA adduct formation. After feeding with olive oil, only a tendency toward reduced adduct formation was noticed. It appears that the type of fatty acids consumed may have an impact on colon cancer risk too [101]. Additionally, research on the impact of MUFA in patients with metabolic syndrome (MetS) supports the notion that postprandial oxidative stress is decreased by the MUFA diet in these patients. These findings suggest that understanding the possible cardioprotective advantages of dietary monounsaturated fat is critical, particularly in people with the MetS.

### 3.3. Impact of Edible Oil Supplements to Oxidative Stress Biomarkers

#### 3.3.1. Fish Oil Supplements

##### Human/Animal Consumption

Studies evaluating the effects of fish oil supplementation to counteract oxidative stress are summarized in Table 3. Rocha et al. [25] examined commercial fish oil in gel capsules rich in omega-3 as a therapeutic intervention for the reduction in plasma triglyceride concentration in male Wistar rats following a hypercholesterolemic diet. Treatment with fish oil had a protective effect against hypercholesterolemic diet in mice models, increasing SOD activity and not affecting lipid peroxidation measuring biochemically (MDA). The role of syntaxin-3, a single molecule effector in cell membrane expansion, is thought to be the mechanism by which omega-3 fatty acids reduce the MDA biomarker. It is also possible that omega-3 fatty acids reduce lipid peroxidation by altering the structure of cellular membranes by stimulating syntaxin 3 [85]. However, the effect of omega-3 FAs supplementation on the reduction in MDA seems to be conducted through changes in the lipid profile composition too. Another study assessed the association between the usage of omega-3 FA supplement during pregnancy and urinary oxidative stress biomarker (8-iso-PGF2α) concentrations [86]. The study showed a decrease in oxidative stress, especially in third trimester pregnancy, associated with lower concentrations of 8-isoPGF2α, suggesting a decrease in maternal oxidative stress during pregnancy.

Perales et al. [7] hypothesized that fish oil consumption may protect against atherosclerotic vascular disease, determining in vitro and in vivo the effects of dietary cholesterol and fish-oil intake on the apoptotic pathways in smooth muscle cells. Fish oil attenuated the increase in apoptotic markers through its influence on the expression of antioxidant genes, indicating that the replacement of a cholesterol-rich diet with a fish oil-rich diet could increase the resistance of stromal cells to apoptosis, counteracting oxidative stress related mechanisms.

**Table 3:** Edible oil used as supplements to counteract oxidative stress.

	Animal Model/Cell Category	Edible Oil	Fatty Acids in Details	Oxidative Stress and Inflammation Biomarkers	Antioxidants	Application	Study	Reference
Marine oils	Male Wistar rats following or not hypercholesterolemic diet	Fish oil commercially available in gel capsules	33.57% of saturated FAs, 30.28% of monounsaturated FAs, 31.1% of n-3 PUFAs, and 3.61% of n-6 PUFAs	malondialdehyde (MDA) levels were not affected for both groups	Increase in Erythrocyte SOD concentration.	Therapeutic intervention for the reduction in plasma triglyceride concentration	In vivo	[27]
	Women enrolled during pregnancy (32.6 weeks)	Omega-3 FA supplements, listed as “fish oil supplements”	Not mentioned	8-iso-PGF2α lower levels of 8-iso-PGF2α associated with n-3 FA intake in pregnancy	Antioxidants were not evaluated	Effect of omega-3 FA consumption during oxidative stress in pregnancy	In vivo	[102]
	In vivo/in vitro cell model was used, culturing SMC isolated from chicks	10% of menhaden oil	Not mentioned	Apoptotic cell death markers	Fish oil attenuated the increase in apoptotic markers through its influence on the expression of antioxidant genes.	Control of cholesterol levels	In vitro and in vivo	[7]
	Healthy adults	Fish oil	60% of omega-3 fatty acids (36% eicosapentaenoic acid [EPA] and 24% docosahexaenoic acid [DHA])	Oxidized low-density lipoprotein (ox-LDL) and lipid peroxidation total antioxidant capacity, glutathione peroxidase, superoxide dismutase	Effect of fish oil against fine particulate air pollution in China	Control air pollution	Clinical trial	[103]
	Healthy young adults	Fish oil	fish-oil capsule contains 60% omega-3 fatty acids (36% EPA and 24% DHA)	Fluorescein-5-thiosemicarbazide to detect carbonyl protein	Total antioxidant activity, glutathione	Biomarkers of skin inflammation and oxidative stress	Clinical trial	[104]
	Healthy young males	Fish oil	Not mentioned	Plasma thiobarbituric acid reactive substances (TBARS) H_2_O_2_ stimulated DNA damage	-	Reduction in selected markers of oxidative stress after a single bout of eccentric exercise	Clinical trial	[105]
	Adult male Wistar rats	Fish oil capsules	N-3 PUFA (both EPA and DHA)	Lipid hydroperoxide	SOD and GPx activities	Effect of fish oil on oxidative stress and inflammation in asthma	In vivo	[88]
	Patients with multiple sclerosis	4 g/day omega Rx capsules	n-3 PUFA (both EPA and DHA)	Lipid peroxidation in serum	-	Efficacy of fish oil in multiple sclerosis patients	Clinical trial	[106]
	Monocyte cells U937 incubated in a high-glucose medium.	Fish oil emulsion	Not mentioned	Protein carbonyls	Antioxidant, superoxide dismutase activity, and isoprostane	Efficacy of fish oil in cells mimicking hyperglycemia	In vitro	[107]
	Healthy adults	Fish oil	1000 mg EPA and 400 mg DHA;	IL-6, IL-1β, IL-8, and TNF-α	-	Effect of fish oil on common markers of systemic inflammation	Clinical trial	[108]
	Large yellow croaker	Fish oil	Not mentioned	Lipid peroxidation,	Antioxidant enzyme activity	Effects of oxidized dietary lipids on growth performance of large yellow croaker	In vivo	[109]
	ALM12 cell line and male C57BL/6J mice	DHA	Not mentioned	*Intracellular ROS Detection*	-	Effect of DHA to protect hepatocytes from oxidative damage	In vitro and in vivo	[8]
	Patients undergoing (CABG) surgery	N-3 polyunsaturated fatty acids	N-3 polyunsaturated fatty acids	Total peroxides, endogenous peroxidase activity	-	Effect of post-operative oxidative stress in the course of CABG surgery	Clinical trial	[100]
	HIV-seropositive Patients	Omega-3 fatty acid ethyl esters	Omega-3 fatty acid ethyl esters	Lipid peroxidation products	Glutathione levels	Effect of omega-3 fatty acid in HIV-seropositive patients	Clinical trial	[110]
	Patients with multiple sclerosis	High-dose ω-3 fatty acid	High-dose ω-3 fatty acid	Lipid peroxidation,	-	Effect of high-dose ω-3 fatty acids in patients with multiple sclerosis	Clinical trial	[111]
	Children with attention deficit hyperactivity disorder	N-3 fatty acids 635 mg eicosapentaenoic acid (EPA), 195 mg docosahexaenoic acid (DHA)	-	-	Activity of glutathione reductase (GR), catalase (CAT) and superoxide dismutase (SOD)	Effect of n-3 fatty acids in children with attention deficit hyperactivity disorder	Clinical trial	[18]
	Patients with Alzheimer’s disease	Omega-3 fatty acids	DHA (22:6) and 0.6 g EPA (20:5)	-	2-isoprostane, 8-iso-PGF2α,	Effect of Omega-3 fatty acids in patients with Alzheimer’s	Clinical trial	[112]
	Patients with Prostate cancer	Fish oil	-	Oxidative phosphorylation	-	Effect of fish oil in Patients with Prostate cancer	Clinical trial	[113]
	Patients on hemodialysis	N-3 PUFA and soybean oil	1.28 g/day of n-3 PUFA	Oxidation protein products	Isoprostanes, vitamins C and E, total antioxidant capacity	Effect of n-3 PUFA and soybean oil in patients on hemodialysis	Clinical trial	[114]
Vegetable oils	Peripheral blood mononuclear cells after exercise in young athletes	Almond and olive oil	Not mentioned	Lipid peroxidation, protein carbonyl derivatives and nitrotyrosine	Vitamin E	Effects of dietary almond- and olive oil on athletic performance	In vitro	[115]
	Hepatocytes	Olive oil	Phenolic compounds	Reactive oxygen species	Not mentioned	Influenced the outcome of cell responses in conditions of increased oxidative stress	In vitro	[116]
	Intestinal cells	Olive oil polyphenols	Not mentioned	H_2_O_2_ production, IL-6 and IL-8 release	Glutathione (GSH)	H_2_O_2_ production, GSH decrease, IL-6 and IL-8 release	In vitro	[117]
	Lens, skin, and serum of Sprague-Dawley male albino rats	Flax seed oil (FSO)	Not mentioned	MDA	(GSH) levels (GPx) superoxide dismutase (SOD) activities	Ultraviolet C exposure led to oxidative stress and FSO can protect	In vivo	[118]

#### 3.3.2. Vegetable, Essential Oils Supplements

##### Human/Animal Studies

Sesame oil is regarded as a natural sunscreen. Sesamol, a phenolic antioxidant, suppresses UVB-induced ROS generation, lowers lipid peroxidation, and thiobarbituric acid reactive substances in skin cells. It was demonstrated that sesamol affects the specifically modulating oxidative enzyme myeloperoxidase (MPO) and other proteins, which are detrimental to human well-being [76]. Moreover, sesame oil offers cardioprotection due to the molecular mechanism of this food ingredient, attributed to the methylenedioxy group present in the sesamol component [76]. Flax seed oil (FSO) (*Linum usitatissimum* L.), a different vegetable oil, demonstrated photoprotective effects against ultraviolet C-induced apoptosis and oxidative stress in rats [118]. Moreover, olive oil contained phenolic compounds which appeared to protect cells against induced oxidative stress (H_2_O_2_-induced)-related damage and to be able to modulate redox signaling by chelating intracellular labile iron [116]. Furthermore, olive oil in intestinal cells promoted a reduction in oxysterols, affecting the imbalance and pro-inflammatory response of the cells [117]. Therefore, phytochemicals containing high levels of antioxidants can play a key role in cancer chemoprevention by suppressing oxidative stress-induced DNA damage [46,119]. On the other hand, Eder et al. claim that neither the diet based on rapeseed oil, which includes linolenic acid, nor the diet based on olive oil, which mostly contains oleic acid, provided any appreciable protective advantage against oxidative DNA damage in the livers of female rats. It was also shown that a diet high in the linoleic acid-rich sunflower oil can increase erythrocyte oxidative stress, cause tissue microinjury, and generate a larger phagocyte oxidative burst than consuming the same amount of energy from saturated fatty acids and n-3 PUFA derived from marine sources. This was discovered after analyzing the effect of dietary fats on the oxidative–antioxidative status of blood in rats. At the same caloric level, rats given sunflower oil exhibited higher plasma antioxidant activity than rats given other fats [120]. Regarding other oil types [121], a cross-sectional study comparing the consumption of coconut oil and sunflower oil among people with coronary artery disease in India found that those who used coconut oil had better antioxidant status than those who used sunflower oil, with lower levels of vitamin C and a higher rate of lipid peroxidation, both indicators of increased oxidative stress.

The beneficial effect of microalgae oil with comparable efficacies to fish oil for protection from cardiovascular risk factors was observed by lowering plasma triglycerides and oxidative stress biomarkers in clinical trials [88]. Furthermore, magnolia flower essential oil, isolated from the flowers, presented antibacterial activity, due to higher free radical scavenging, and antioxidant activity compared to other tested essential oil (bud oil) [122].

## 4. Conclusions

The interaction of various cell types with fish oils, vegetable oils, or essential oils enhances the biological data from a biochemical and safety perspective. As a result, this knowledge enables the identification and characterization of potential molecular targets and active biomarkers involved in a variety of nutritional disorders, including fatty liver disorders, diabetes mellitus, disorders of protein metabolism, iron deficiency anemia, and dyslipidemias. Additionally, it is anticipated that the use of biocompatibility assays could be advantageous for offering more precise therapies that advise patients to consume particular fatty acids according to their demands (personalized diet) and the particular cell type of interest, as well as for safety considerations. The scant amount of the literature available, however, makes it evident that further research in these areas is still needed before precision nutrition may be used in clinical settings and public health contexts all over the world. In conclusion, public health will be improved in terms of food and drug administration if more fundamental studies are performed to examine the cell interactions with various potential edible oils.

## Data Availability

The data presented in this study are available online.
